# CEA (CEACAM5) expression is common in muscle‐invasive urothelial carcinoma of the bladder but unrelated to the disease course

**DOI:** 10.1002/bco2.354

**Published:** 2024-04-02

**Authors:** Henning Plage, Kira Furlano, Jörg Neymeyer, Sarah Weinberger, Benedikt Gerdes, Mandy Hubatsch, Bernhard Ralla, Antonia Franz, Annika Fendler, Michela de Martino, Florian Roßner, Simon Schallenberg, Sefer Elezkurtaj, Martina Kluth, Maximilian Lennartz, Niclas C. Blessin, Andreas H. Marx, Henrik Samtleben, Margit Fisch, Michael Rink, Krystian Kaczmarek, Thorsten Ecke, Steffen Hallmann, Stefan Koch, Nico Adamini, Sarah Minner, Ronald Simon, Guido Sauter, Joachim Weischenfeldt, Tobias Klatte, Thorsten Schlomm, David Horst, Henrik Zecha, Marcin Slojewski

**Affiliations:** ^1^ Department of Urology Charité – Universitätsmedizin Berlin, Corporate Member of Freie Universität Berlin, Humboldt‐Universität zu Berlin and Berlin Institute of Health Berlin Germany; ^2^ Institute of Pathology Charité – Universitätsmedizin Berlin, Corporate Member of Freie Universität Berlin, Humboldt‐Universität zu Berlin and Berlin Institute of Health Berlin Germany; ^3^ Institute of Pathology University Medical Center Hamburg‐Eppendorf Hamburg Germany; ^4^ Department of Pathology Academic Hospital Fuerth Fuerth Germany; ^5^ Department of Urology University Medical Center Hamburg‐Eppendorf Hamburg Germany; ^6^ Department of Urology Marienhospital Hamburg Hamburg Germany; ^7^ Department of Urology and Urological Oncology Pomeranian Medical University Szczecin Poland; ^8^ Department of Urology Helios Hospital Bad Saarow Bad Saarow Germany; ^9^ Department of Pathology Helios Hospital Bad Saarow Bad Saarow Germany; ^10^ Department of Urology Albertinen Hospital Hamburg Germany; ^11^ Biotech Research & Innovation Center (BRIC) University of Copenhagen Copenhagen Denmark; ^12^ Finsen Laboratory Rigshospitalet Copenhagen Denmark

**Keywords:** bladder cancer, CEA, CEACAM5, immunohistochemistry, prognosis, tissue microarray

## Abstract

**Objectives:**

Carcinoembryonic antigen (CEA) is a cell surface glycoprotein that represents a promising therapeutic target. Serum measurement of shedded CEA can be utilized for monitoring of cancer patients.

**Material and Methods:**

To evaluate the potential clinical significance of CEA expression in urothelial bladder neoplasms, CEA was analysed by immunohistochemistry in more than 2500 urothelial bladder carcinomas in a tissue microarray format.

**Results:**

CEA staining was largely absent in normal urothelial cells but was observed in 30.4% of urothelial bladder carcinomas including 406 (16.7%) with weak, 140 (5.8%) with moderate, and 192 (7.9%) with strong staining. CEA positivity occurred in 10.9% of 411 pTaG2 low‐grade, 32.0% of 178 pTaG2 high‐grade, and 43.0% of 93 pTaG3 tumours (*p* < 0.0001). In 1335 pT2–4 carcinomas, CEA positivity (34.1%) was lower than in pTaG3 tumours. Within pT2–4 carcinomas, CEA staining was unrelated to pT, pN, grade, L‐status, V‐status, overall survival, recurrence free survival, and cancer specific survival (*p* > 0.25).

**Conclusion:**

CEA increases markedly with grade progression in pTa tumours, and expression occurs in a significant fraction of pT2–4 urothelial bladder carcinomas. The high rate of CEA positivity in pT2–4 carcinomas offers the opportunity of using CEA serum measurement for monitoring the clinical course of these cancers. Moreover, CEA positive urothelial carcinomas are candidates for a treatment by targeted anti‐CEA drugs.

## 1 INTRODUCTION

Urinary bladder cancer is the 10th most common malignant tumour type worldwide.^1^ About 80% of patients present with low‐grade non‐invasive (pTa) or minimally invasive (pT1) stage cancers, which are characterized by a good prognosis and can be removed by transurethral resection. However, because almost all of these tumours recur and about 20% will further progress to muscle‐invasive disease,^2^ regular follow‐up examination at close intervals is mandatory. In patients with muscle‐invasive bladder cancer, treatment usually consists of neoadjuvant chemotherapy plus radiotherapy or radical cystectomy, but outcomes remain variable and almost 50% of the patients develop early metastasis and eventually die from their disease.^3^ It is hoped that a better understanding of the molecular features driving urothelial carcinoma will eventually enable a better prediction of the individual patient prognosis, optimize treatment decisions, and enable the development of efficient new cancer drugs.

Carcinoembryonic antigen (CEA; CEACAM5) is a cell adhesion glycoprotein localized on the cell surface.^4^ During fetal development, CEA is extensively expressed in many tissues, but expression ceases in most tissues immediately before birth.^5^ In adults, CEA is especially expressed in non‐keratinizing squamous epithelium, colorectal epithelium, and surface epithelium of the stomach mucosa. Overexpression of CEA is found in various cancer types (summarized in the literature^6^, ^7^, ^8^, ^9^). CEA is a promising drug target. For example, the antibody‐drug conjugate tusamitamab ravtansine (tusa) that selectively targets CEA is currently being tested in a phase III study for the treatment of metastatic non‐small cell lung cancer (NCT04154956). An application to the US Food and Drug Administration (FDA) for approval is expected to take place in 2024.^10^ The bispecific antibody cibisatamab (binds to CD3 and CEA) has antitumour activity with radiological shrinkage in 11% of 36 patients treated with cibisatamab monotherapy and in 50% of 10 patients treated with cibisatamab and PD‐L1‐inhibiting antibodies on metastatic CEA positive colorectal carcinomas in a recent phase I study.^11^, ^12^ As CEA protein is released into the blood, its measurement in the blood serum is used for tumour detection and monitoring of disease.^13^, ^14^ Studies on CEA expression in urothelial bladder carcinoma were performed on cohorts of 8 to 1208 patients and have provided controversial results. For example, reported CEA positivity rates ranged from 32% to 100% in muscle‐invasive urothelial carcinoma.^15^, ^16^ One of these studies has suggested a link between high CEA expression and poor prognosis in pT1‐4 urothelial carcinoma.^17^

To learn more on CEA expression in bladder cancer and its potential prognostic role, we took advantage of our large cohort of urothelial bladder carcinomas previously collected within our consortium.^18^ In this study, the relationship between CEA immunostaining and clinicopathological parameters of disease progression as well as patient outcome was analysed in more than 2700 urothelial bladder carcinomas in a tissue microarray (TMA) format.

## 2 MATERIALS AND METHODS

### 2.1 Tissue microarrays (TMA)

The TMA method allows the analysis of a large number of molecular‐genetic alterations on one TMA set. The TMAs used in this study were first employed in a recent study on the prognostic role of GATA3 expression in bladder cancer.^18^ These TMAs contained one sample each from 2710 urothelial tumours of the bladder archived at the Institute of Pathology, University Hospital Hamburg, G4ermany, Institute of Pathology, Charité Berlin, Germany, Department of Pathology, Academic Hospital Fuerth, Germany, or Department of Pathology, Helios Hospital Bad Saarow, Germany, and/or treated at Department of Urology, University Hospital Hamburg, Germany, Department of Urology, Charité Berlin, Germany, Department of Urology, Helios Hospital Bad Saarow, Germany, Department of Urology, Albertinen Hospital, Hamburg, Germany, and Department of Urology and Urological Oncology, Pomeranian Medical University, Szczecin, Poland. Patients at each centre were treated according to the guidelines at the time. In brief, patients with pTa/pT1 disease underwent a transurethral resection of the bladder tumour with or without postoperative or adjuvant instillation therapy, while patients with pT2‐pT4 disease were treated by radical cystectomy. Available histopathological data including grade, tumour stage (pT), lymph node status (pN), and status of venous (V) and lymphatic (L) invasion are shown in Table 1. Clinical follow up data (overall survival; OS: time between cystectomy and death, cancer‐specific survival; CSS: time between cystectomy and cancer‐specific death, recurrence‐free survival; RFS: time between cystectomy and disease recurrence) were available from 636 patients with pT2–4 carcinomas treated by cystectomy (median: 15 months; range: 1–176 months). The tissues were fixed in 4% buffered formalin and then embedded in paraffin. The TMA manufacturing process has previously been described in detail.^19^, ^20^ In brief, one tissue spot (diameter: 0.6 mm) per patient was used. The use of archived remnants of diagnostic tissues for TMA manufacturing, their analysis for research purposes, and patient data were according to local laws (HmbKHG, §12), and analysis had been approved by the local ethics committee (Ethics Commission Hamburg, WF‐049/09). All work has been carried out in compliance with the Declaration of Helsinki.

**TABLE 1:** Patient cohort.

	Study cohort on TMA (*n* = 2710)
Follow up months	636
Months	
Mean	26.7
Median	15.0
Pathological tumour stage	
pTa	887 (39.2%)
pT2	462 (20.4%)
pT3	615 (27.2%)
pT4	298 (13.2%)
Tumour grade	
G2	820 (30.6%)
G3	1858 (69.4%)
Pathological lymph node status	
pN0	734 (62.0%)
pN+	449 (38.0%)
Resection margin status	
R0	595 (80.6%)
R1	143 (19.4%)
Lymphatic vessel infiltration	
L0	275 (49.5%)
L1	281 (50.5%)
Blood vessel infiltration	
V0	450 (74.4%)
V1	155 (25.6%)

*Note*: Per cent in the column “study cohort on TMA” refers to the fraction of samples across each category. Numbers do not always add up to 2710 in the different categories because of cases with missing data.

### 2.2 Immunohistochemistry

For this study, we used identical methods for immunohistochemical evaluation of CEA as previously described (manuscript submitted). Freshly prepared TMA sections were immunostained on 1 day in one experiment. Slides were deparaffinized with xylol, rehydrated through a graded alcohol series, and exposed to heat‐induced antigen retrieval for 5 min in an autoclave at 121°C in pH 9.0 Dako Target Retrieval Solution (Agilent, Santa Clara, CA, USA; #S2367). Endogenous peroxidase activity was blocked with Dako REAL PeroxidaseBlocking Solution (Agilent, Santa Clara, CA, USA; #S2023) for 10 min. Primary antibody specific against CEA protein (rabbit recombinant, MSVA‐465R, MS Validated Antibodies, Hamburg, Germany; #2563‐465R) was applied at 37°C for 60 min at a dilution of 1:150. Bound antibody was visualized using the Dako REAL EnVision Detection System Peroxidase/DAB+, Rabbit/Mouse kit (Agilent, Santa Clara, CA, USA; #K5007) according to the manufacturer's directions. The sections were counterstained with haemalaun. For each tumour tissue, the percentage of CEA positive tumour cells was estimated, and the staining intensity was semi‐quantitatively recorded (0, 1+, 2+, and 3+). For statistical analyses, the staining results were categorized into four groups as follows: Negative: no staining at all, weak staining: staining intensity of 1+ in ≤70% or staining intensity of 2+ in ≤30% of tumour cells, moderate staining: staining intensity of 1+ in >70%, staining intensity of 2+ in >30% but in ≤70% or staining intensity of 3+ in ≤30% of tumour cells, strong staining: staining intensity of 2+ in >70% or staining intensity of 3+ in >30% of tumour cells.

### 2.3 Statistics

Statistical calculations were performed with JMP 16 software (SAS, Cary, NC, USA). Contingency tables and the *χ* ^2^ test were performed to search for associations between CEA immunostaining, other molecular parameters and tumour phenotype. Survival curves were calculated according to Kaplan–Meier. The log‐rank test was applied to detect significant differences between groups. A *p*‐value of ≤0.05 was considered as statistically significant.

## 3 RESULTS

### 3.1 Technical issues

Of our 2710 urothelial bladder carcinomas, 2429 (89.6%) were interpretable for CEA. Non‐informative cases were caused by a lack of unequivocal tumour cells on the TMA spots or absence of entire tissue spot on the TMA.

### 3.2 CEA in urothelial carcinomas

**FIGURE 1:**
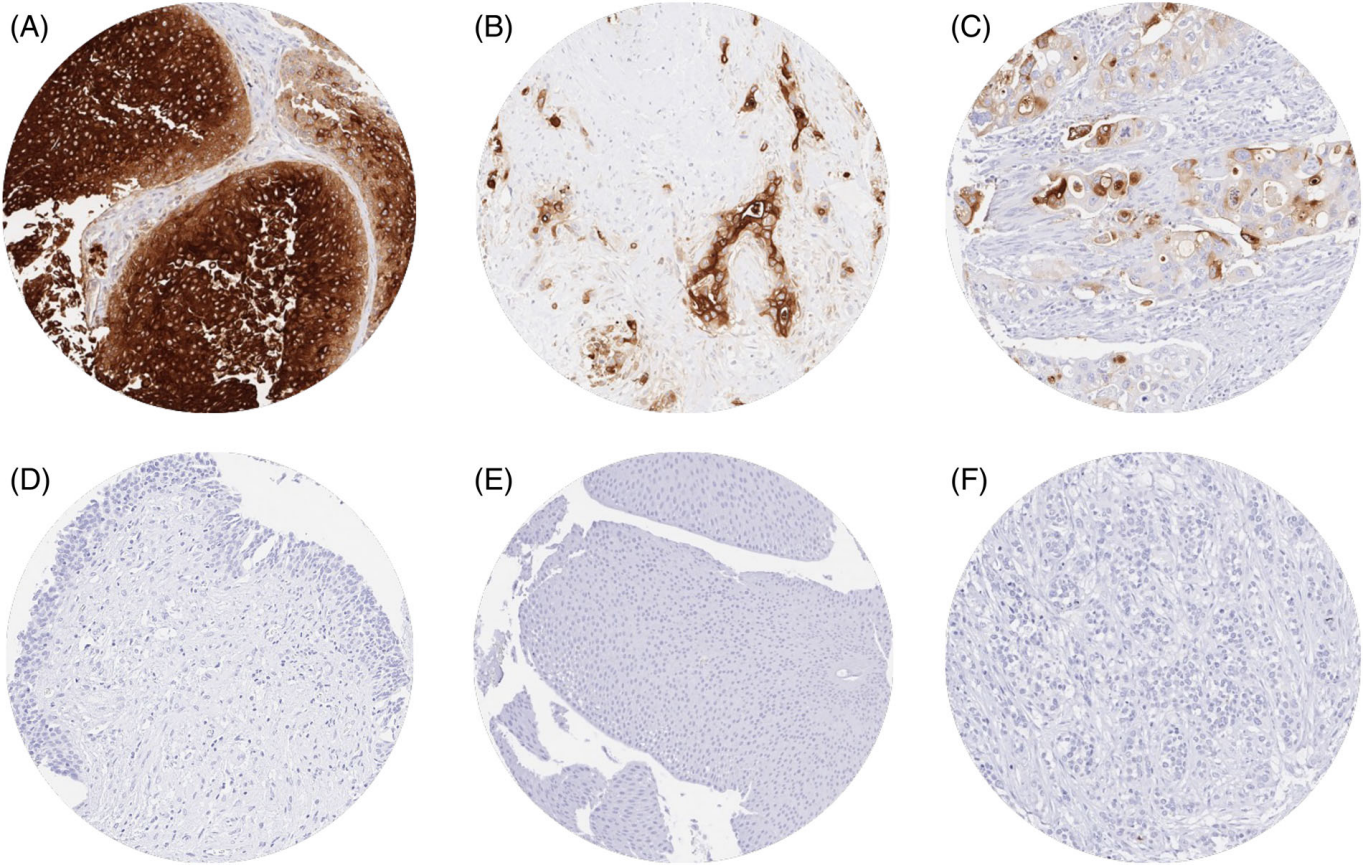
CEA immunostaining in normal and neoplastic urothelium. Cytoplasmic CEA staining is strong in the vast majority of tumour cells in a pTa tumour (A) and a muscle‐invasive urothelial carcinoma (B) while the CEA staining only involves a smaller subset of tumour cells in another pT2 carcinoma (C). CEA staining is absent in normal urothelium (D) as well as in other examples of pTa (E) and muscle‐invasive urothelial carcinomas of the urinary bladder (F).

**FIGURE 2:**
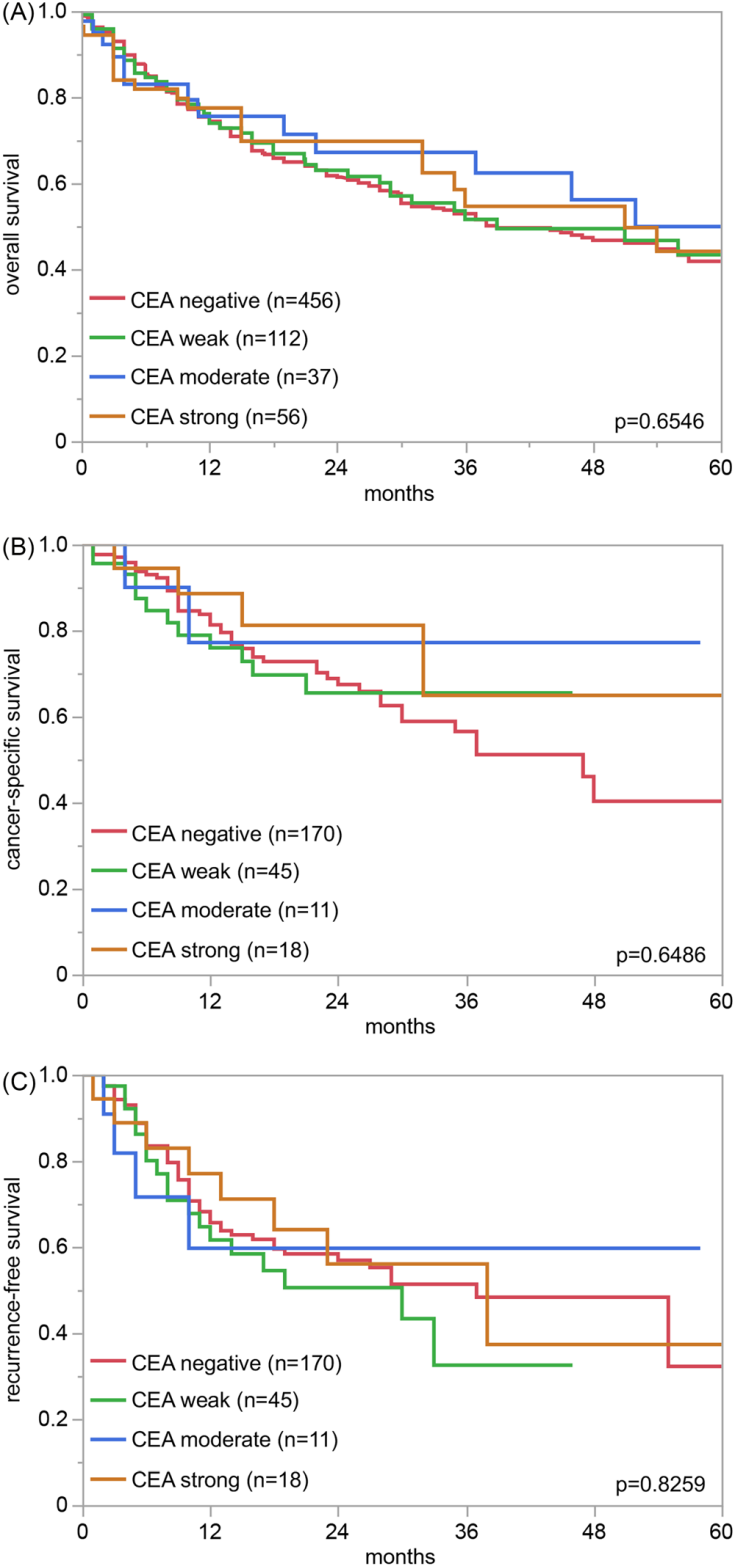
CEA immunostaining and patient prognosis. (A) Overall survival, (B) cancer‐specific survival, and (C) recurrence‐free survival.

CEA staining was largely absent in normal urothelial cells. A cytoplasmic CEA positivity was observed in 738 (30.4%) of urothelial bladder carcinomas including 406 (16.7%) with weak, 140 (5.8%) with moderate, and 192 (7.9%) with strong staining. Representative images of CEA positive and negative cancers and normal tissues are shown in Figure 1. The relationship between CEA staining and tumour phenotype is shown in Table 2. Within pTa tumours, the rate of CEA positivity increased sharply from pTaG2 low‐grade (10.9% positive) to pTaG2 high‐grade (32.0% positive) and pTaG3 (43.0%; *p* < 0.0001). The CEA positivity rate in muscle‐invasive cancers (34.1%) was comparable to the findings in pTaG2 high‐grade tumours but lower than in pTaG3 tumours (*p* = 0.19). Within 1747 pT2–4 carcinomas, CEA immunostaining was unrelated to pT, pN, grade, L‐, and V‐status. In 661 patients with pT2–4 carcinomas who were treated by cystectomy, the CEA staining intensity was not associated with overall, tumour specific or recurrence‐free survival (Figure 2A–C) This also hold true if subsets of pT2, pT3 and pT4 cancers were separately analysed (data not shown).

**TABLE 2:** CEA immunostaining and cancer phenotype.

	*n*	CEA immunostaining result			Strong (%)	*p* value
		Negative (%)	Weak (%)	Moderate (%)		
All cancers	2429	69.6	16.7	5.8	7.9	
pTa G2 low	411	89.1	8.0	1.9	1.0	<0.0001
pTa G2 high	178	68.0	19.1	9.6	3.4	
pTa G3	93	57.0	24.7	9.7	8.6	
pT2	444	67.6	18.2	5.0	9.2	0.7743^a^
pT3	598	66.6	16.4	6.5	10.5	
pT4	293	68.3	16.4	4.4	10.9	
G2	105	60.0	17.1	6.7	16.2	0.1941^a^
G3	1202	68.1	16.9	5.2	9.7	
pN0	667	69.9	15.1	5.1	9.9	0.1179^a^
pN+	440	65.2	19.3	7.0	8.4	
R0	557	70.7	14.9	5.2	9.2	0.3798^a^
R1	139	63.3	20.1	5.8	10.8	
L0	264	72.0	14.0	4.5	9.5	0.2635^a^
L1	274	65.0	20.1	5.1	9.9	
V0	430	70.2	15.3	4.4	10.0	0.2687^a^
V1	151	61.6	19.2	6.6	12.6	

Abbreviations: G, grade; L, lymphatic invasion; pN, pathological lymph node status; pT, pathological tumour stage; R, resection margin status; V, venous invasion.

^a^ Only in pT2–4 urothelial carcinoma.

## 4 DISCUSSION

At least 10 earlier studies had analysed CEA expression by IHC in cohorts of 8 to 56 pT2–4 urothelial bladder carcinomas and described positivity rates of 32% to 100%.^15^, ^16^ Reasons for these discrepant results most likely include the use of different antibodies, staining protocols and criteria for the definition of positivity. Our result of 34% CEA positive pT2–4 cancers is in the lower range of previous studies and very similar to the 30% that we previously observed in a partially overlapping set of pT2–4 carcinomas in a study on over 15 000 tumours from 120 different tumour entities (manuscript submitted). The high intra‐laboratory consistency of data is enabled by a consistent staining interpretation and a highly validated staining approach. Our assay for CEA analysis has previously been validated according to the guidelines of the international working group for antibody validation (IWGAV)^21^ by comparison with a second independent antibody and with RNA expression data obtained from three different publicly accessible databases in 76 different normal tissue categories (manuscript submitted).

Our successful analysis of 682 non‐invasive urothelial carcinomas revealed a marked increase from pTaG2 low‐grade (11%) to pTaG2 high‐grade (32%) and pTaG3 tumours (43%). This is in contrast to data from one previous study where pTa tumours of different grades were analysed by CEA IHC and CEA positivity was reported in 32% of 50 pTaG2 and 2% of 50 pTaG3 tumours.^22^ The marked and continuous increase of CEA expression with grade in non‐invasive urothelial bladder carcinomas is consistent with a role of CEA expression during tumour progression but the lower rates of of CEA positivity in pT2–4 carcinomas than in pTaG3 and the complete lack of further clinico‐pathological association for CEA expression in muscle‐invasive carcinomas appear to contradict an active tumour promoting role of CEA. The counterintuitive relationship of CEA expression with grade and stage may be caused by the unique evolution of pTa bladder cancers in vivo. Non‐invasive urothelial neoplasms tend to diffusely disseminate within the bladder and the upper urinary tract.^23^ Resection of papillary tumours is thus often incomplete and clonally related tumour remnants frequently remain in the bladder as invisible flat lesions or minor papillary tumours, which serve as a source for a multitude of subsequent recurrences. Similarly, as tumour cell lines in vitro, non‐invasive urothelial neoplasms can thus continuously accumulate genomic alterations over a long period of time.^24^ In many patients, pTa tumour evolution is only terminated if the neoplastic cells acquire the capability of invasive tumour growth, which may eventually terminate genomic tumour progression by the cancer related death of the patient. As in other tumour entities, the accumulation of genomic alterations results in an increasing degree of cellular atypia and aberrant expression of a continuously growing number of genes in high‐grade tumours.^25^, ^26^

The “histologic grade” is by default higher in pTaG3 tumours than in pT2–4 carcinomas that also include tumours with G2 morphology. Accordingly, several studies have described higher rates of genomic alterations in pTaG3 than in pT2–4 carcinomas.^24^, ^27^ A link of CEA expression with cellular dedifferentiation is also consistent with the lack of clinically relevant associations of CEA expression in pT2–4 carcinomas. In this tumour category, tumours with rather benign morphology such as nested type carcinomas are prone to a similarly poor prognosis as high‐grade carcinomas.^28^ Given the lack of prognostic impact of grade in muscle‐invasive urothelial carcinoma, it was recommended to avoid grading in these tumours in the WHO classification of genitourinary carcinomas since 2004.^29^ Considering that molecular features with an unequivocal functional role in cancer cells such as p53 alterations,^30^ HER2 overexpression,^31^ and MYC^32^ have also failed to be prognostic in most studies on pT2–4 bladder cancer demonstrates that the absence of a prognostic relevance of the expression of a specific gene in urothelial cancer does rule out a relevant role in tumour biology.

Although CEA IHC is obviously not a suitable prognostic marker in urothelial carcinoma, CEA positivity in these tumours may have relevant diagnostic and therapeutic implications. Considering the high CEA expression in many tumour entities and the rather limited CEA levels in vital normal tissues (manuscript submitted), CEA constitutes an attractive therapeutic target. Initial attempts of targeting CEA by humanized anti‐CEA antibodies have led to disappointing results, probably because a shedding of CEA from tumour cells caused a binding of therapeutic antibodies to circulating CEA in the blood stream, which prevented these antibodies from reaching the tumour cells (summarized in Gold et al.^33^). Current approaches use CEA as a target for antibody‐drug conjugates^10^, ^12^ (NCT04154956), vaccination (summarized in Turriziani et al.^34^) and CAR‐T cells (summarized in Lei et al.^35^). More than 200 phase I and II clinical trials are currently ongoing for CEA targeting drugs. These studies involve colorectal, oesophageal, stomach, gastric, lung, breast and pancreatic carcinomas or CEA positive tumours irrespective of their sites of origin (www.ClinicalTrials.gov). An application to the FDA for approval of the antibody‐drug conjugate tusamitamib ravtansine (tusa) for the treatment of metastatic non‐small cell lung cancer is expected in 2024.^10^ Based on our data, it might be well possible that a significant percentage of bladder cancer patients could benefit from tusa or other anti‐CEA drugs.

Most of the clinical interest in CEA came earlier from its role as a serum marker for colorectal cancer (CRC). In CRC, serial CEA serum measurements is recommended in postoperative surveillance of stage II and III patients who may be candidates for surgical resection or systemic therapy in the event of distant metastasis and for monitoring of therapy in advanced disease.^36^ Our data show that comparable levels of tumoural CEA expression can occur in a fraction of urothelial bladder carcinomas. As almost 100% of CRCs show high‐level CEA expression, serological CEA monitoring does not require CEA analysis in tumour tissue. It appears possible that a serological serum screening could also be useful for disease and therapy monitoring in these urothelial cancer patients with documented high‐level expression of CEA. Multiple studies have earlier demonstrated a clinical utility of CEA serum measurement in patients with gastric,^37^ pancreatic,^38^ non‐small cell lung,^39^ and breast cancer^40^ although most of these studies have not optimized patient selection by immunohistochemical CEA analysis of tumour tissue.

In summary, these data show that CEA is expressed in a significant fraction of pT2–4 urothelial carcinomas. Although CEA positivity is unrelated to cancer aggressiveness, the high rate of CEA positive cases offers the opportunity of using CEA serum measurement for follow‐up controls of CEA positive cancers. Moreover, CEA positive urothelial carcinomas are candidates for a treatment by targeted anti‐CEA drugs.

## AUTHOR CONTRIBUTIONS

Henning Plage, Marcin Slojewsk, Martina Kluth, Ronald Simon, Thorsten Schlomm and Guido Sauter contributed to conception, design, data collection, data analysis and manuscript writing. Henning Plage, Maximilian Lennartz, Niclas C. Blessin, Sarah Minner, Florian Roßner, Simon Schallenberg, Sefer Elezkurtaj, Andreas H. Marx, Henrik Samtleben, Stefan Koch and David Horst participated in pathology data analysis and data interpretation. Kira Furlano, Henning Plage, Steffen Hallmann, Sarah Weinberger, Bernhard Ralla, Antonia Franz, Annika Fendler, Michela de Martino, Florian Roßner, Simon Schallenberg, Sefer Elezkurtaj, Andreas H. Marx, Henrik Samtleben, Margit Fisch, Michael Rink, Marcin Slojewsk, Krystian Kaczmarek, Thorsten Ecke, Steffen Hallmann, Stefan Koch, Nico Adamini, Tobias Klatte, David Horst and Henrik Zecha collected the samples. Ronald Simon and Martina Kluth contributed to data analysis. Henning Plage, Marcin Slojewsk, Thorsten Schlomm, Ronald Simon and Guido Sauter did the study supervision. All authors agree to be accountable for the content of the work.

## CONFLICT OF INTEREST STATEMENT

The rabbit recombinant CEA antibody, clone MSVA‐465R was obtained from MS Validated Antibodies GmbH, Hamburg, Germany (owned by a family member of GS).

## Data Availability

All data generated or analysed during this study are included in this published article.
